# miR-130a can predict response to temozolomide in patients with glioblastoma multiforme, independently of O6-methylguanine-DNA methyltransferase

**DOI:** 10.1186/s12967-015-0435-y

**Published:** 2015-02-21

**Authors:** Huiyuan Chen, Xinyi Li, Wenbin Li, Huyong Zheng

**Affiliations:** Cancer Center, Beijing Shijitan Hospital, Capital Medical University, Beijing, 100038 China; Hematology Oncology Center, Beijing Key Laboratory of Pediatric Hematology Oncology, National Key Discipline of Pediatrics, Beijing Children’s Hospital, Capital Medical University, Beijing, 100045 China; University of South Florida, Tampa, FL 33612 USA

**Keywords:** Glioblastoma, Temozolomide, MicroRNAs

## Abstract

**Background:**

Currently, O6-methylguanine-DNA methyltransferase(MGMT) promoter methylation is the most convincing predictive biomarker for temozolomide (TMZ) response in patients with glioblastoma multiforme (GBM). However, technical obstacles prevent this biomarker from being applied widely. On the other hand, microRNAs (miRNAs) are easily investigated in the clinical setting using quantitative real-time polymerase chain reactions. This study aimed to identify miRNAs that could serve as predictive biomarkers for TMZ response.

**Methods:**

The Cancer Genome Atlas (TCGA) and the Chinese Glioma Genome Atlas (CGGA) databases were used to investigate the significance of associations between miRNA expression and overall survival (OS) in TMZ-treated patients with GBM. Cytotoxicity assays were used to validate the miRNAs’ roles in the response of glioma cells to TMZ. Biological insights concerning the miRNAs were explored using gene set enrichment analysis (GSEA) and gene ontology (GO) analysis.

**Results:**

miR-130a was found to be significantly associated with OS in TMZ-treated patients from TCGA and the CGGA. In contrast, miR-130a appeared to be unassociated with OS in patients who only received radiotherapy. The TMZ cytotoxicity assay showed that miR-130a over-expression could sensitize response to TMZ in glioma cells. GSEA and GO analysis indicated that lower miR-130a could generate a more extensive response to oxidative stress, which in turn could elevate Ape1 and mediate resistance to TMZ. *In vitro* experiment verified that cells with lower miR-130a express higher Ape1 under oxidative stress.

**Conclusions:**

Our data suggested that miR-130a could be a predictive marker for TMZ response in patients with GBM, independently of the mechanism by which MGMT acts as a biomarker. miR-130a could serve as a guide for treatment strategy selection in cases of GBM.

## Background

Glioblastoma multiforme (GBM) is the most malignant and common brain tumor in adults. Temozolomide (TMZ) is currently the first-line chemotherapy for this disease. TMZ can cross the blood–brain barrier and significantly improves the prognosis of patients with GBM [1]. However, survival remains poor among these patients because of resistance to TMZ. Biomarkers have been developed to predict patients’ responses to TMZ.

O6-methylguanine-DNA methyltransferase (MGMT) promoter methylation is currently the most convincing predictive biomarker for TMZ response, based on its correlation with overall survival (OS) in patients with GBM who have received TMZ chemotherapy [2-4]. However, this biomarker remains unsuitable for guiding GBM treatment in widespread application because of technical obstacles [4].

MicroRNAs (miRNAs) are small non-coding RNAs that are more stable than messenger RNAs. Since quantitative real-time polymerase chain reaction (qRT-PCR) is widely used to quantify RNA expression levels in clinical practice, it would be convenient to have a miRNA that could be used to predict response to TMZ treatment in patients with GBM. Previous studies [5-11] reported several kinds of miRNAs which have predictive value for TMZ response in glioblastoma. These studies mainly focused on one special target of a miRNA. That may not explain the whole function of the miRNA, since one kind of miRNA can regulate hundreds of genes. Bioinformatic analyses, on the other hand, could illustrate the miRNA function from the macroscopic view. In this study, using bioinformatic methods, we sought to identify such a miRNA, finding evidence that miR-130a is correlated with OS in TMZ-treated patients with GBM. Further, the biological process involving miR-130a is independent of MGMT.

## Material and methods

### Datasets

The Cancer Genome Atlas (TCGA) database [12] included miRNA expression profiling of 184 adult patients who had GBM and underwent radiation therapy and TMZ chemotherapy. Data on these patients were used as a discovery set. We downloaded the mRNA expression profiling and clinical information on these patients, as well as the miRNA expression profiling of another 128 glioblastoma patients in TCGA who only received radiation therapy. Additionally, we obtained miRNA expression profiling of 28 adult patients who had glioblastoma and underwent TMZ chemotherapy from the Chinese Glioma Genome Atlas (CGGA) database [13]. Data on these 28 patients were used as a validation set.

### Cell culture

Human glioma cell lines U87, LN229, SHG44, and U251 were cultured in DMEM/F-12 culture medium (Gibco, Life Technologies, Carlsbad, CA, USA) supplemented with 10% fetal bovine serum (Gibco). Cells were maintained in a humidified incubator with 5% CO_2_ at 37°C. The All-in-One miRNA qRT-PCR Detection Kit (FulenGen, Guangzhou, China) was used to evaluate the miR-130a levels in cells.

### Transfection

Regarding miR-130a mimics and control miRNA, inhibitors for miRNA-130a and controls were purchased from FulenGen in China. Transfection was performed using Lipofectamine RNAiMAX Transfection Reagent (Invitrogen, Life Technologies, Carlsbad, CA, USA), following the manufacturer’s instructions. Transfection efficiency was also evaluated using the All-in-One miRNA qRT-PCR Detection Kit.

### TMZ cytotoxicity assay

After transfection, cells were seeded at a density of 6000 cells per well on 96 well plates in triplicates. TMZ (Sigma Aldrich, St. Louis, MO, USA) was added at various concentrations ranging from 0 to 1000 μmol/L for 48 hours. The Cell Titer 96 AQueous One Solution Cell Proliferation Assay kit (Promega, Madison, WI, USA) was used to determine the number of viable cells in the TMZ cytotoxicity assay. To test the cleavage of nuclear poly (ADP-ribose) polymerase (PARP), proteins of SHG44 and U251 were extracted after transfection and 96 hours 500 μM TMZ treatment.

### Induction of Oxidative Stress with hydrogen peroxide

At 48 hours after transfection, cells were washed twice with PBS, then incubated in complete medium supplemented with 100 μM hydrogen peroxide to induce oxidative stress. The incubation continued for 24 hours before determination of Ape1 expression using western blotting.

### Western Blotting

Proteins were extracted using RIPA buffer (Cell Signaling Technology) supplemented with protease inhibitor. Proteins were separated by 10% SDS-PAGE and transferred to PVDF membrane. After blocking with 5% skim milk in TBST, the membranes were incubated with primary antibodies overnight at 4°C. The membranes were then washed and incubated with secondary antibodies for 1 hour. Antibody binding was assessed by enhanced chemiluminescence substrate (BioRad). Antibodies to PARP and Ape1 were purchased from Proteintech in China.

### Statistical analysis

For each miRNA, the association between the expression level and OS in the discovery set was calculated using a univariate Cox proportional hazards regression analysis. The 4 miRNAs that were found to be significantly associated with OS in the discovery set were then evaluated in the validation set using the Kaplan-Meier method and 2-sided log-rank tests. Subsequently, the miRNA that was significantly associated with OS in both sets was further analyzed using a Cox multivariate analysis that included O-6-methylguanine-DNA methyltransferase (MGMT) methylation status, age, and gender as covariates. The association between the expression of this miRNA and OS was also investigated in the group of patients from TCGA who had received radiation therapy alone. As previously, the Kaplan-Meier method and 2-sided log-rank tests were also used in this analysis. The cutoff value for this miRNA in TCGA was defined as the median expression level.

The miRNA-associated gene set was identified using a Spearman correlation analysis. The negatively correlated genes were included in a gene ontology (GO) analysis that was performed using DAVID [14]; (National Institute of Allergy and Infectious Diseases, Bethesda, MD, USA). Additionally, we performed a Gene Set Enrichment Analysis (GSEA) with GSEA software downloaded from the Broad Institute [15,16] (Cambridge, MA, USA).

Paired-sample t-tests were employed to assess the significance of differences between TMZ cytotoxicity assay results.

Statistical analyses of TCGA and CGGA datasets were performed using BRB-Array Tools 4.3.0 Beta_2, which was developed by Dr. Richard Simon and the BRB-Array Tools Development Team (National Cancer Institute, Bethesda, MD, USA). Statistical analyses for *in vitro* experimental results were performed using SPSS 16.0 (IBM SPSS, Inc., Chicago, IL, USA). Two-sided p-values less than 0.05 were regarded as statistically significant.

## Results

### miR-130a was correlated with overall survival in TMZ-treated patients with GBM, but not in non-TMZ-treated patients

**Figure 1:**
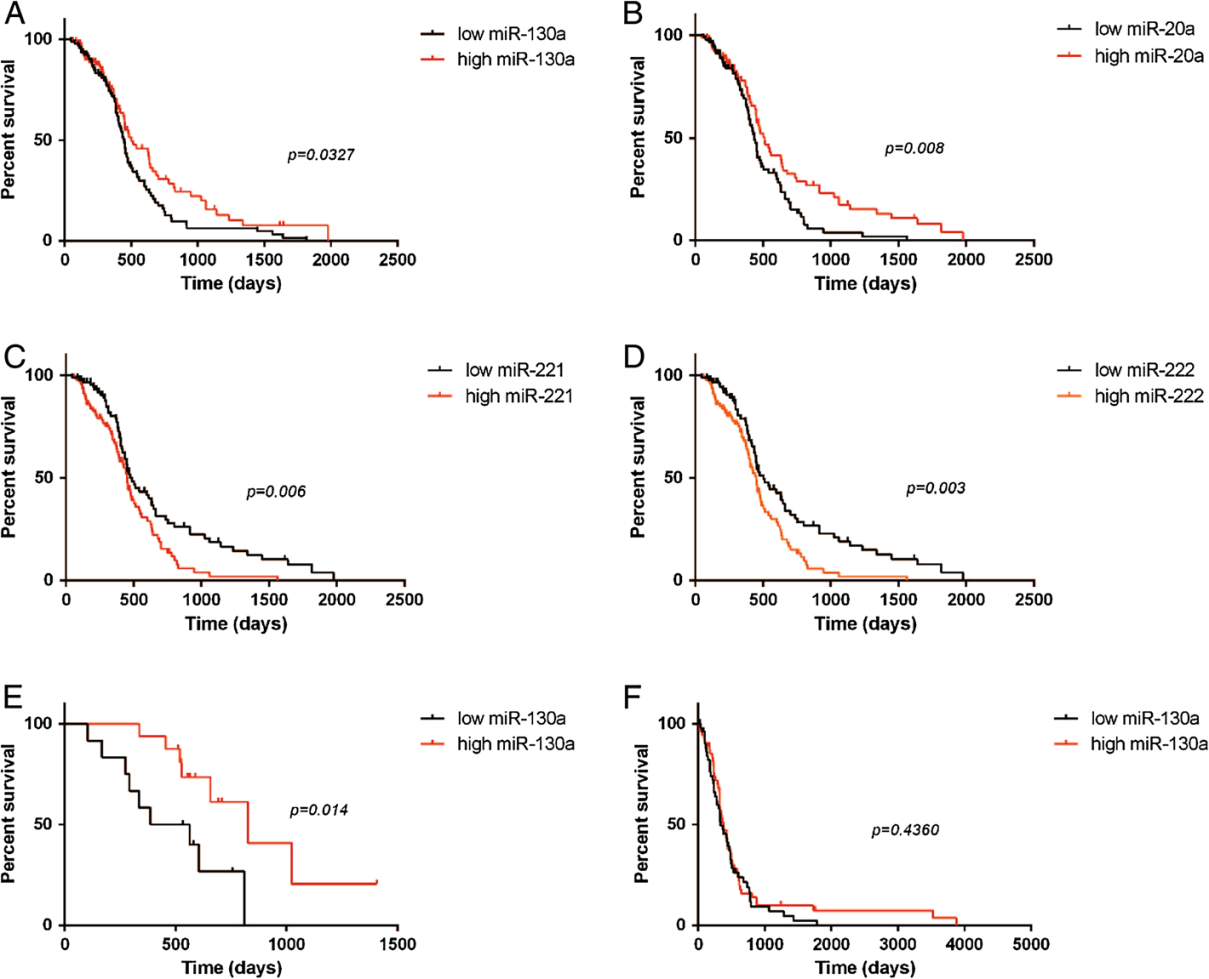
**Kaplan-Meier estimates of overall survival. (A)** Higher miR-130a expression level was associated with improved survival in temozolomide (TMZ)-treated glioblastoma multiforme(GBM) patients from The Cancer Genome Atlas (TCGA). **(B)** Higher miR-20a expression level was associated with improved survival in TMZ-treated GBM patients from TCGA. **(C)** Higher miR-221 expression level was associated with decreased survival in TMZ-treated GBM patients from TCGA. **(D)** Higher miR-222 expression level was associated with decreased survival in TMZ-treated GBM patients from TCGA. **(E)** Higher miR-130a expression level was associated with improved survival in TMZ-treated GBM patients from the Chinese Glioma Genome Atlas. **(F)** The miR-130a level was not correlated with overall survival in patients from TCGA who did not receive treatment with TMZ.

Cox univariate analyses showed that miR-130a, miR-20a, miR-221, and miR-222 were correlated with OS in TMZ-treated patients with GBM from TCGA (Table 1, Figure 1A,B,C and D). These 4 miRNAs were then evaluated in the validation dataset using the Kaplan-Meier method and 2-sided log-rank tests. miR-130a was found to be significantly correlated with OS in both of the datasets (Figure 1E).

**Table 1:** **miRNAs correlated with OS of patients treated with temozolomide in TCGA**

**Variable**	**Hazard ratio**	**95% CI**	***p value***
miR-130a	0.558	0.426–0.733	*2.48 ∙ 10* ^*−5*^
miR-222	1.341	1.166–1.541	*3.65 ∙ 10* ^*−5*^
miR-221	1.416	1.187–1.690	*0.0001093*
miR-20a	0.693	0.560–0.857	*0.0007161*

CI, confidence interval.

MGMT methylation status, age, and gender were analyzed using univariate Cox analyses. MGMT methylation status and age were found to be significantly associated with OS (Table 2). miR-130a was then analyzed in the group of TMZ-treated GBM patients from TCGA using a multivariate Cox analysis that included MGMT methylation status and age as covariates (Table 2). miR-130a remained significantly associated with OS in this analysis.

**Table 2:** **Cox regression analyses of the associations of miR-130a and clinical characteristics with OS in TCGA**

**Variable**	**Univariate Cox regression**			**Multivariate Cox regression**		
	**Hazard ratio**	**95% CI**	***p value***	**Hazard ratio**	**95% CI**	***p value***
Gender	0.776	0.536–1.125	*0.181*			
Age	1.027	1.012–1.042	*0.001*	1.016	0.998–1.035	*0.088*
MGMT methylation	0.559	0.361–0.864	*0.009*	0.654	0.413–1.008	*0.054*
miR-130a	0.558	0.426–0.733	*0.000*	0.665	0.473–0.935	*0.019*

CI, confidence interval; MGMT, O-6-methylguanine-DNA methyltransferase.

Further, the Kaplan-Meier method and 2-sided log-rank tests showed that miR-130a was not significantly associated with OS in the patients who only received radiation therapy (Figure 1F).

### Biological insights regarding miR-130a

**Figure 2:**
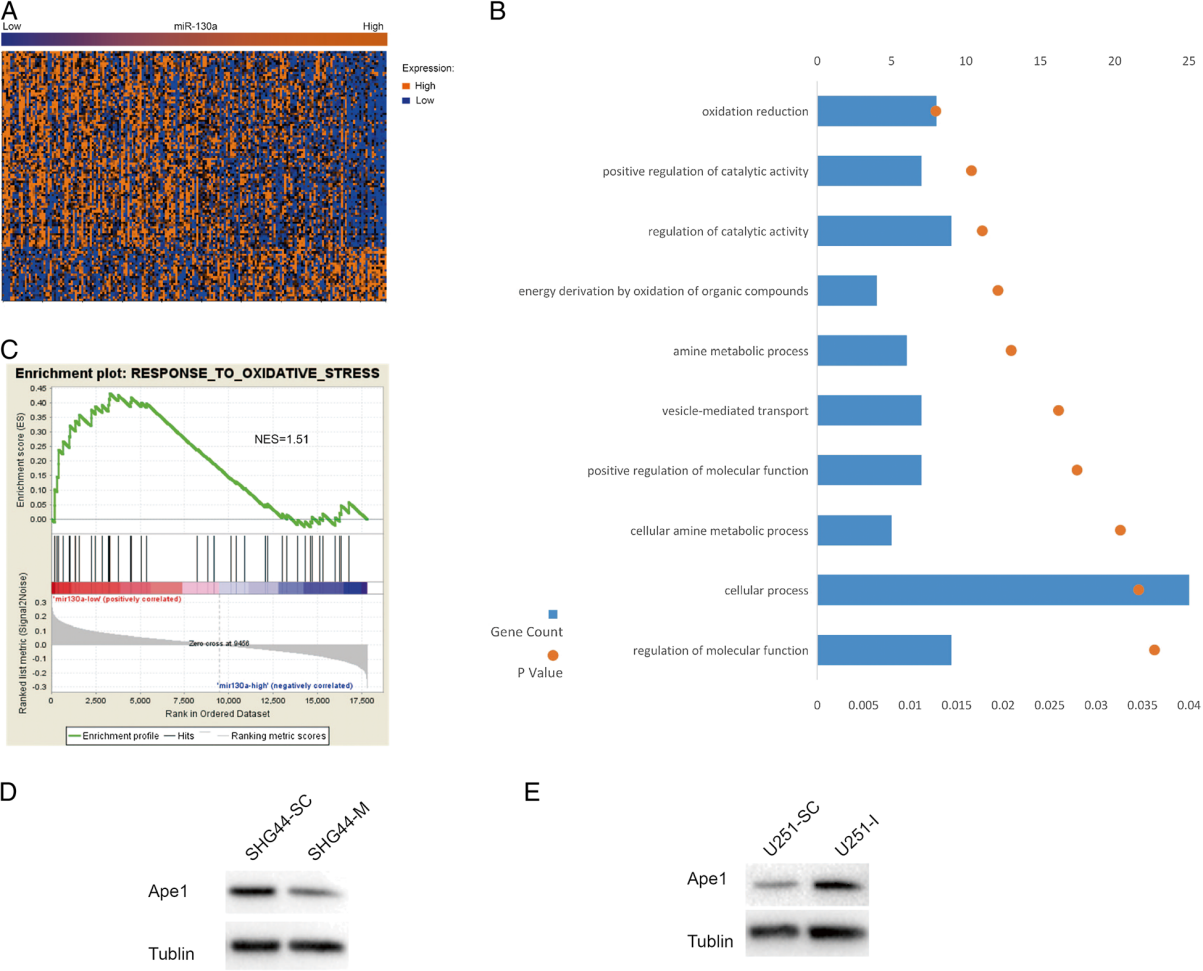
**Biological insights regarding miR-130a. (A)** Heat map of the gene expression signature correlated with miR-130a expression. Columns represent patients and rows represent probes. Patients are ordered from left to right by increasing miR-130a expression. **(B)** The top 10 gene ontology terms for miR-130a negatively associated genes. **(C)** Gene set enrichment analysis showed enrichment of genes related to response to oxidative stress among patients with low miR-130a expression. NES, normalized enrichment score. **(D)** SHG44 transfected with the scrambled control (SHG44-SC) expressed higher Ape1 than cells transfected with miR-130a mimics (SHG44-M) did under oxidative stress. **(E)** U251 transfected with the scrambled control (U251-SC) expressed lower Ape1 than cells transfected with miR-130a inhibitors (U251-I) did under oxidative stress.

GO analysis and GSEA were conducted to provide biological insights concerning the role of miR-130a. The mRNA expression profiling data were used to find miR-130a-correlated genes, as assessed using Spearman’s correlation coefficient. The eighty-six genes (Figure 2A) that were negatively correlated with miR-130a were then included in GO analyses. The top 10 GO terms are presented in Figure 2B. As shown in the figure, miR-130a-related genes had a relatively tight association with oxidation reduction.

In addition, GSEA showed enrichment of genes related to response to oxidative stress (ROS) among patients with low miR-130a expression (normalized enrichment score [NES] =1.51, p = 0.037).

**Figure 3:**
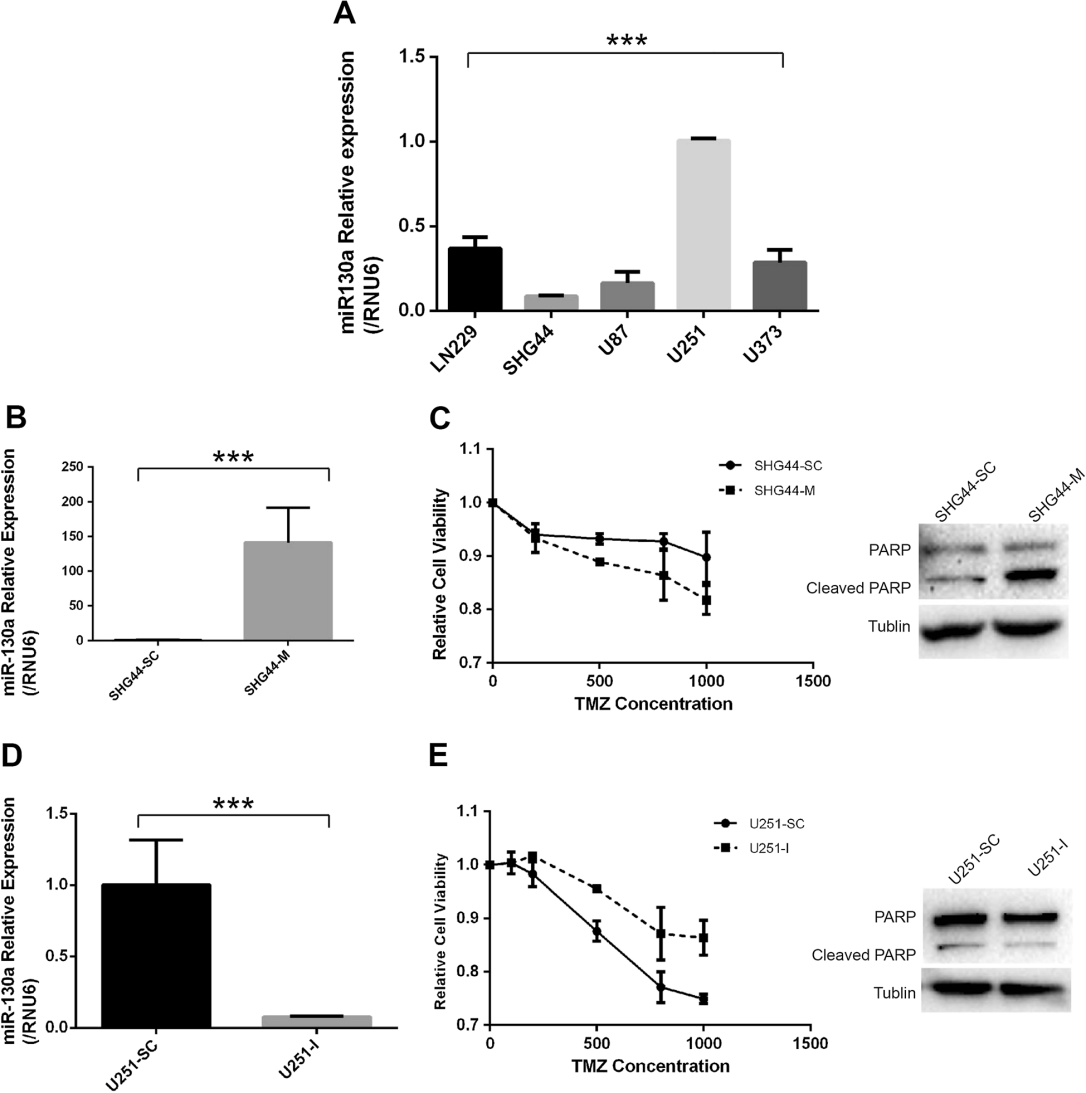
**miR-130a sensitized the response of glioma cells to temozolomide (TMZ). (A)** miR-130a level in 5 glioblastoma multiforme(GBM) cell lines. **(B)** SHG44 was transfected with miR-130a mimics (SHG44-M) and scrambled control miRNAs (SHG44-SC). **(C)** SHG44-M were more sensitive to TMZ than SHG44-SC (*p = 0.027*). Overexpression of miR-130a increased PARP cleavage in SHG44 induced by TMZ. **(D)** U251 was transfected with inhibitors for miR-130a (U251-I) and the scrambled control (U251–SC). **(E)** U251-I were more resistant to TMZ than U251–SC (*p = 0.039*). Knockdown of miR-130a decreased PARP cleavage in U251 induced by TMZ.

The miR-130a levels were relatively low in SHG44 cells (Figure 3A). This cell line was then transfected with miR-130a mimics and the scrambled control (Figure 3B). Western blotting showed that SHG44 transfected with the scrambled control (SHG44-SC) expressed higher Ape1 than cells transfected with miR-130a mimics (SHG44-M) did under oxidative stress (Figure 2D).

The miR-130a levels were relatively high in U251 cells (Figure 3A). This cell line was then transfected with inhibitors (5′-AUGCCCUUUUAACAUUGCACUG-3′) for miR-130a and the scrambled control (Figure 3D). Western blotting showed that U251 transfected with the scrambled control (U251-SC) expressed lower Ape1 than cells transfected with miR-130a inhibitors (U251-I) did under oxidative stress (Figure 2E).

### miR-130a over expression sensitized the response of glioma cells to TMZ

The TMZ cytotoxicity assay showed that SHG44-M were more sensitive to TMZ than SHG44-SC (p = 0.027, Figure 3C). Western blotting showed that overexpression of miR-130a increased PARP cleavage in SHG44 induced by TMZ (Figure 3C).

U251-I were found to be more resistant to TMZ than U251-SC (p = 0.039, Figure 3E). Western blotting showed knockdown of miR-130a decreased PARP cleavage in U251 induced by TMZ (Figure 3E).

## Discussion

In our univariate Cox analyses of TCGA data, miR-130a, miR-20a, miR-221, and miR-222 showed significant associations with the OS of patients with GBM who received TMZ chemotherapy. However, only the predictive capacity of miR-130a was validated by an analysis of CGGA data on patients with GBM who received TMZ chemotherapy. In a further analysis of TCGA data, we also observed that miR-130a failed to correlate with the OS of patients with GBM who received radiation therapy alone.

The role of miR-130a in the chemo-resistance of GBM cells was also determined in the present study. SHG44 with up-regulated miR-130a showed higher sensitivity than parental cells. On the other hand, U251 with down-regulated miR-130a showed lower sensitivity than parental cells. The cytotoxicity of TMZ was less than 50% in these investigations because of the short duration of treatment. Further *in vitro* functional research on miR-130a is necessary.

On the whole, higher miR-130a expression was associated with prolonged OS in patients who had GBM and received TMZ chemotherapy. miR-130a could be a candidate predictive biomarker for TMZ response.

miR-130a has been reported to reduce resistance to Gefitinib and TNF-related apoptosis-inducing ligand (TRAIL) in Non-small cell lung cancer by down-regulating MET and miRNA-221/222 [17,18]. To investigate the biological mechanism of miR-130a in GBM, we performed GSEA and GO analysis of miR-130a-associated genes. Oxidation reduction and ROS were showed to be involved in miR-130a-correlated biological processes.

MGMT promotes clinical resistance to chemotherapy through its ability to remove the O6-methylguanine (O6-meG) adduct produced by TMZ [19,20]. In fact, TMZ also generates several other kinds of DNA adducts in cells. N7-methylguanine (7-meG) and N3-methyladenine (3-meA) are the most abundant of these DNA adducts [4]. They are precursors of abasic sites, which can impede DNA replication [21-23]. The base excision repair (BER) pathway is responsible for repairing these lesions. Apurinic/apyrimidinic endonuclease 1 (Ape1) is the key enzyme in BER pathways that remove abasic sites induced by TMZ [24,25]. The function of Ape1, which is independent of MGMT, can lead to cell resistance to TMZ treatment [26]. A small-molecule inhibitor of Ape1 has been found to block proliferation and reduces viability of glioblastoma cells [27]. It has been reported that oxidative stress could elevate Ape1, which in turn mediates chemoresistance by repairing abasic sites produced by TMZ [28,29].

**Figure 4:**
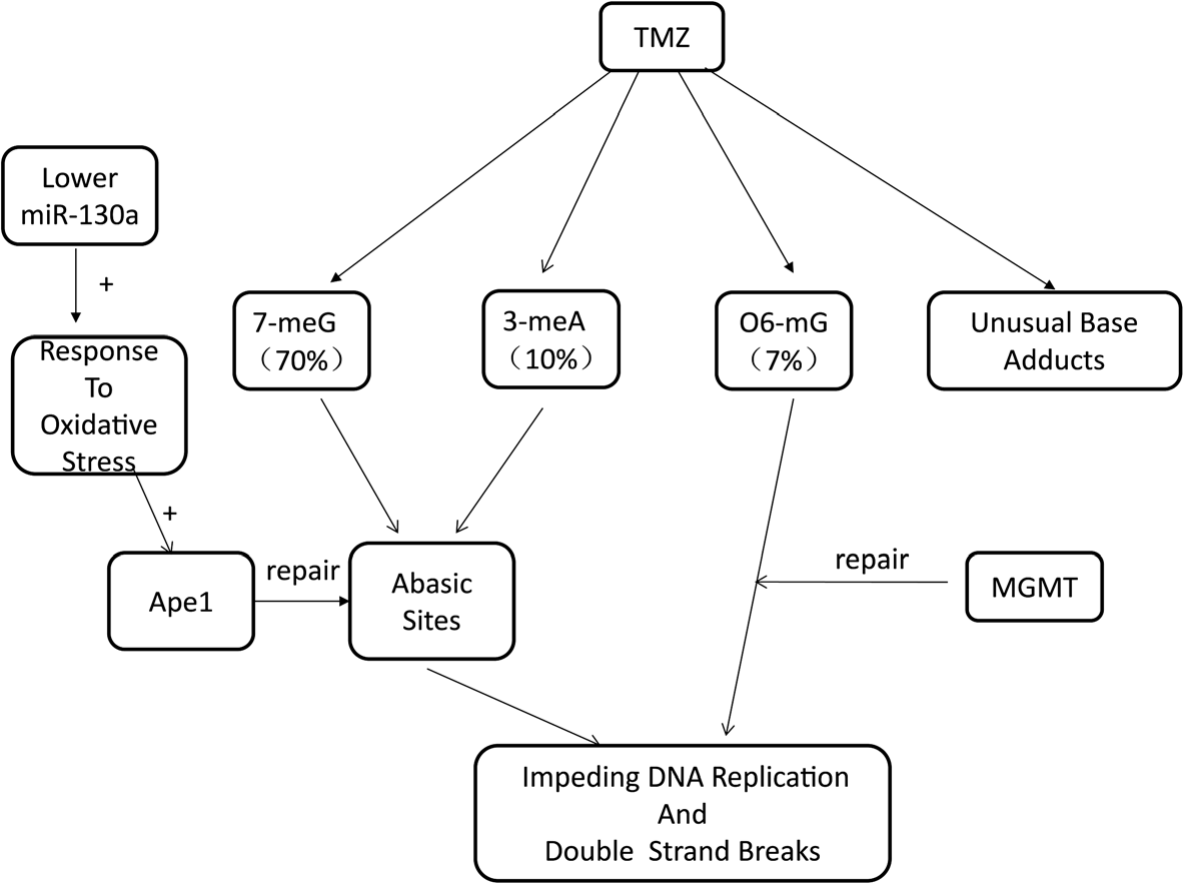
**Lower miR-130a could generate more extensive response to oxidative stress, which in turn could elevate Ape1 and the repair of abasic sites. In double-stranded DNA, N7-methylguanine (7-meG), N3-methyladenine (3-meA) and O6-methylguanine (O6-meG) accounts for approximately 70, 10, and 7% of temozolomide (TMZ)-induced DNA adducts, respectively. O6-meG adduct is repaired by O6-methylguanine-DNA methyltransferase (MGMT).** 7-meG and 3-meA could generate abasic sites, which can impede DNA replication. Apurinic/apyrimidinic endonuclease 1 (Ape1) is the key enzyme that removes abasic sites. Patients with lower miR-130a could generate a more extensive response to oxidative stress, which in turn could elevate Ape1 and the repair of abasic sites, finally mediating resistance to TMZ.

GO analysis and GSEA showed a negative correlation between response to oxidation stress and miR-130a. Patients with lower miR-130a expression possess a higher ability to respond to oxidative stress. In the present study, we found that cells with lower miR-130a could express higher Ape1 under oxidative stress. Several factors could induce oxidative stress in glioma, including seizures [30], angiogenesis associated nitric oxide [31], surgery associated inflammation, and radiotherapy-delivered reactive oxygen species. We hypothesize that, in the presence of these factors, patients with lower miR-130a could generate a more extensive response to oxidative stress, which in turn could elevate Ape1 and the repair of abasic sites, finally mediating resistance to TMZ (Figure 4). Because Ape1-repaired DNA adducts are different from O6-mG, this process is independent of MGMT. It appears that miR-130a was an even better predictive marker than MGMT methylation in the present study, since the former was significantly correlated with OS in a multivariate Cox analysis, while the latter was not (Table 2).

## Conclusions

In summary, we have reported that miR-130a could be a predictive marker for TMZ response in patients with GBM, and that this predictive ability is independent of the predictive ability of MGMT. Further, the miR-130a biomarker is more convenient to test than MGMT methylation. Its application could provide guidance when selecting treatment strategies for GBM in the future.
